# Maternal stress induced anxiety-like behavior exacerbated by electromagnetic fields radiation in female rats offspring

**DOI:** 10.1371/journal.pone.0273206

**Published:** 2022-08-23

**Authors:** Ehsan Hosseini, Mahsa Farid Habibi, Shirin Babri, Gisou Mohaddes, Hajar abkhezr, Hamed Heydari

**Affiliations:** 1 Faculty of Veterinary Medicine, Division of Physiology, Department of Basic Science, Urmia University, Urmia, Iran; 2 Drug Applied Research Center, Tabriz University of Medical Sciences, Tabriz, Iran; 3 Neuroscience Research Center, Tabriz University of Medical Sciences, Tabriz, Iran; 4 Department of Physiology, School of Medicine, Tabriz University of Medical Sciences, Tabriz, Iran; University of Illinois at Urbana-Champaign, UNITED STATES

## Abstract

There is a disagreement on whether extremely low frequency electromagnetic fields (ELF-EMF) have a beneficial or harmful effect on anxiety-like behavior. Prenatal stress induces frequent disturbances in offspring physiology such as anxiety-like behavior extending to adulthood. This study was designed to evaluate the effects of prenatal stress and ELF-EMF exposure before and during pregnancy on anxiety-like behavior and some anxiety-related pathways in the hippocampus of female rat offspring. A total of 24 female rats 40 days of age were distributed into four groups of 6 rats each: control, Stress (rats whose mothers underwent chronic stress), EMF (rats whose mothers were exposed to electromagnetic fields) and EMF/S (rats whose mothers were simultaneously exposed to chronic stress and ELF-EMF). The rats were given elevated plus-maze and open field tests and then their brains were dissected and their hippocampus were subjected to analysis. ELISA was used to measure 24(S)-hydroxy cholesterol, corticosterone, and serotonin levels. Cryptochrome2, steroidogenic acute regulatory protein, 3B-Hydroxy steroid dehydrogenase, N-methyl-D-aspartate receptor 2(NMDAr2) and phosphorylated N-methyl-D-aspartate receptor 2(PNMDAr2) were assayed by immunoblotting. Anxiety-like behavior increased in all treatment groups at the same time EMF increased anxiety induced by maternal stress in the EMF/S group. The stress group showed decreased serotonin and increased corticosterone levels. ELF-EMF elevated the PNMDAr2/NMDAr2 ratio and 24(S)-hydroxy cholesterol compared to the control group but did not change corticosterone. EMF did not restore changes induced by stress in behavioral and molecular tests. The results of the current study, clarified that ELF-EMF can induce anxiety-like behavior which may be attributed to an increase in the PNMDAr2/NMDAr2 ratio and 24(S)-OHC in the hippocampus, and prenatal stress may contribute to anxiety via a decrease in serotonin and an increase in corticosterone in the hippocampus. We also found that anxiety-like behavior induced by maternal stress exposure, is exacerbated by electromagnetic fields radiation.

## 1. Introduction

Stress can be described as a situation in which homeostasis is altered by numerous reactions, due to stress factors [1]. One of the most prevalent types of stresses is prenatal, which can exert emotional, behavioral and cognitive changes in offspring [2–6]. Anxiety is a behavioral change that introduces enormous problems in the social life of offspring in adulthood [7,8]. Current studies on anxiety are typically in male animals, while female animals are often excluded because of interference of estrogen [9–11]. In fact, there are reports that women are twice as likely as men to experience anxiety disorders and depression [12,13], Because of this, we selected female rats to study changes in anxiety behavior. It has been found that maternal stress can have persistent effects on the neurodevelopment of offspring, such as rate of neurotransmitter secretion and receptors [14]. On the other hand, extremely low frequency electromagnetic fields (ELF-EMF) (ELF 3–3000 Hz) [15] are prevalent in daily life, bringing about an escalating concern with regard to their potential harmful effects [16,17]. We chose an electromagnetic field with frequency of 50 Hz because this frequency is produced by most electrical home appliances [18,19].

**Fig 1:**
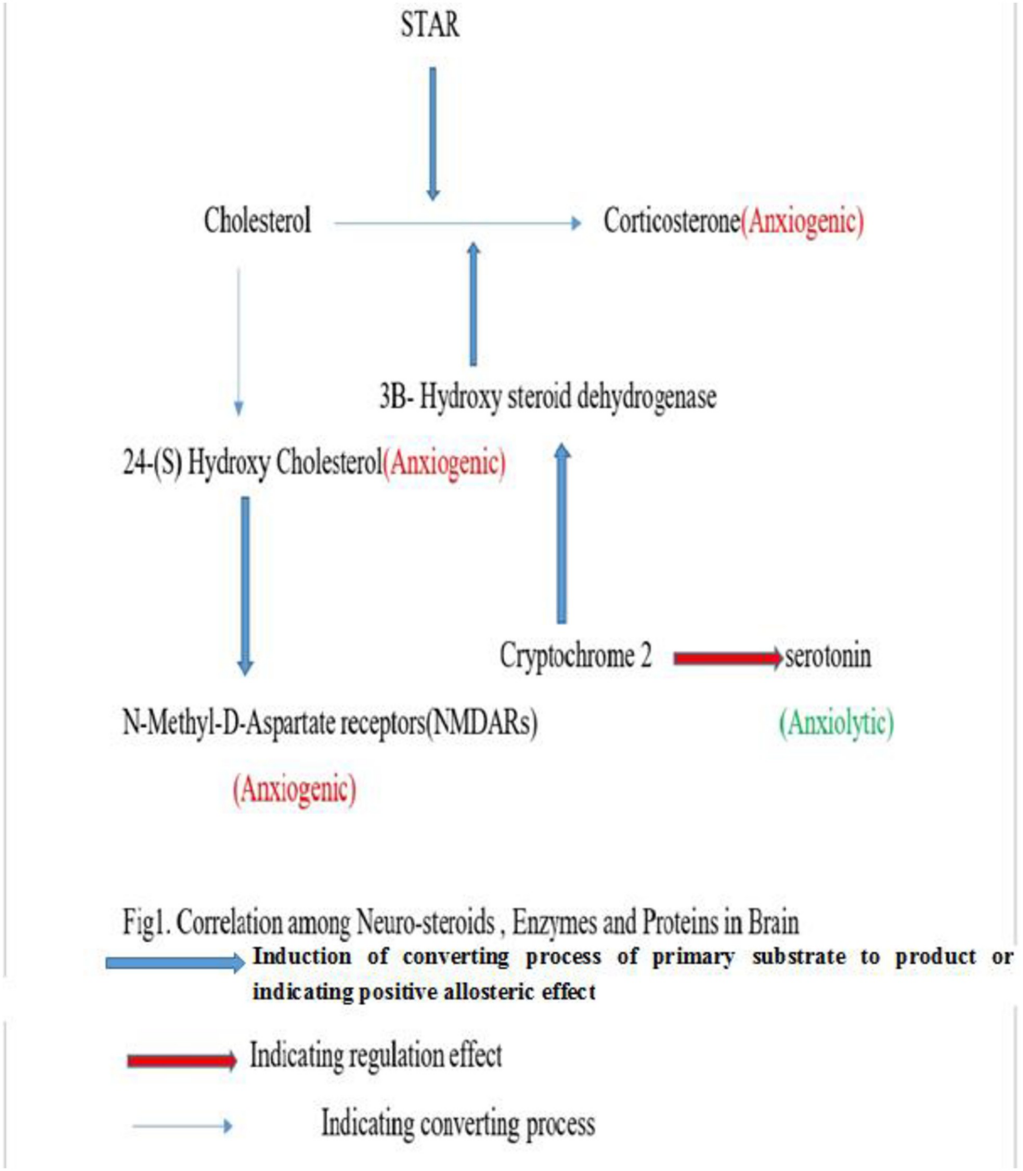
Correlation among neuro-steroids, enzymes and proteins in brain.

According to some studies, ELF-EMF is the same as physiological stress and can cause oxidative stress in the brains of rats [20], chick embryonic cells and humans erythrocytes [21], and kestrel lymphocytes [22]. Numerous studies has been performed on behavior of animal which indicates that ELF-EMF are safe in some cases [23,24] but they are abundant studies declaring of hazardous effect of them in promoting anxiety and depression behaviors in animals [25–27] and human [28,29]. On the other hand, another research has demonstrated some anti-anxiety effects of low frequency electromagnetic fields in mice [30]. Overall, however, most studies show that ELF-EMF increases anxiety-like behavior. Also, there are few studies that addressed the effect of prenatal ELF-EMF exposure on anxiety-like behavior in offspring [26]. Thus, the two factors ELF-EMF and prenatal stress increase the risk of developing anxiety-like behavior, and on the other hand, knowing that these two factors are found in abundance in modern life, how these two factors simultaneously affect anxiety-like behavior is unknown. One view could be that these two factors could have a dual aggravating effect on anxiety-like behavior, and the other view could be that these two factors may interfere with each other’s ability to create anxiety-like behavior, and this could be a way to use ELF-EMF to prevent the harmful effects of prenatal stress. The hippocampus has a clear role in anxiety behavior in animals [31] and several studies have demonstrated that the hippocampus is highly affected by prenatal stress in respect to receptors and neurotransmitters [32–35]. The N-Methyl-D-Aspartate receptor (NMDAr) is an important receptor in the hippocampus involved in anxiety-like behavior [36,37]. Cholesterol is crucial for brain function and neurotransmission [38] because neuroactive steroids (NASs) produced from cholesterol modulate brain processes and interact with diverse receptors such as NMDArs [39]. 24(S)-hydroxy cholesterol is a neuroactive steroid responsible for numerous aspects of brain development and function, such as axon and dendrite growth and synaptogenesis [40], and is a positive allosteric modulator of NMDArs (Fig 1) [41,42]. Another imperative neurostroids is corticosterone, the main corticosteroid hormone in rats, which is associated with a considerable increase in anxiety-like behavior [43] Corticosterone receptors are extremely concentrated in the hippocampus [44]. Steroidogenic acute regulatory protein (STAR) is a key protein that contributes to the production of corticosterone [45] (Fig 1). Moreover, it has been found that stress increases STAR expression in the hippocampus [46]. Furthermore, 3B-Hydroxy steroid dehydrogenase (3B-HSD) is an imperative enzyme in the synthesis process of neurostroids in different brain regions of rats (Fig 1) [47]. Cryptochrome genes, as a part of the circadian cycle system, have a direct effect on behaviors associated with anxiety since circadian clock-deficient cryptochrome knockout mice showed increased anxiety-like behavior [48] and anhedonic-like behavior was observed in cryptochrome-deficient mice [49]. Additionally, mice exhibiting higher anxiety behavior showed lower expression levels of cryptochrome2 in the hippocampus in comparison with normal mice [50]. Serotonin in the hippocampus is involved in anxiety-like behavior [51] and the serotonergic system in hippocampus regulates anxiety-like behavior [52]. Considering that prenatal stress is one of the most common factors in inducing anxiety-like behavior in offspring in modern societies [53–55] and on the other hand due to the confinement of modern living environment with ELF-EMF and the anxiogenic effect of ELF-EMF [25,26,56,57] and also considering the key role of the hippocampus in anxiety-like behavior [31], we hypothesized that if these two factors may have synergic effect of inducing anxiety-like behavior via metabolic changes in hippocampus or the interaction of these two factors can reduce their ability to create anxiety-like behavior. For the first time, to our knowledge, our study simultaneously investigated anxiety-like behavior, neurostroids formation and metabolism, cryptochrome2 expression as a marker of circadian rhythm, glutamate receptor activation, and serotonin production in the hippocampus of female rat offspring which were prenatally stressed and/or exposed to EMF.

## 2. Materials and methods

### 2.1 Animals

Twenty-four female Wistar rats of 3 months of age and weighing 200–250 g were obtained from the Tabriz University Animal Care Center and were kept for one week prior to the study at 25° C and 12 h light and 12 dark conditions for adaptation to the lab environment. During this time, animals had free access to food and water. All procedures and experiments were performed in accordance with the regulations of the Tabriz Medical University Ethical Committee for the protection of animals in research (IR.TBZMED.VCR.REC.1397.230) under the guidelines of the National Institutes of Health.

### 2.2 Experimental design

Female Wistar rats were randomly divided into four groups of 6 rats each (n = 6): control (C), electromagnetic field (EMF), stress (S), and electromagnetic field with stress (EMF-S) groups. Dams in the control group were exposed to a switched-off jammer device. Dams in the EMF group were exposed to ELF-EMF (50 Hz, 100 μT) for 21 days, 4 hours each day from 10:00 a.m. to 14:00 p.m. Dams in the S group were subjected to different types of stressors for 21 days (Table 1). The EMF-S group dams were exposed to ELF-EMF and stressors at the same time for 21 days. After this 21-day period, mating was permitted between male and female rats overnight (each Dam with 2 male rats in a cage). First, the vaginal plaque was checked and after ensuring that the female rat was pregnant, we put the pregnant rat to be treated with second 21-days course of stress (group S) or electromagnetic field (group ELF-EMF) or both stress and electromagnetic field (group EMF/S). All offspring were weaned on day 21 and then female offspring were selected for behavioral tests and measuring the desired indices. Behavioral tests and sampling were performed at the age of 40 days (Flowchart 1).

**Flowchart 1:**
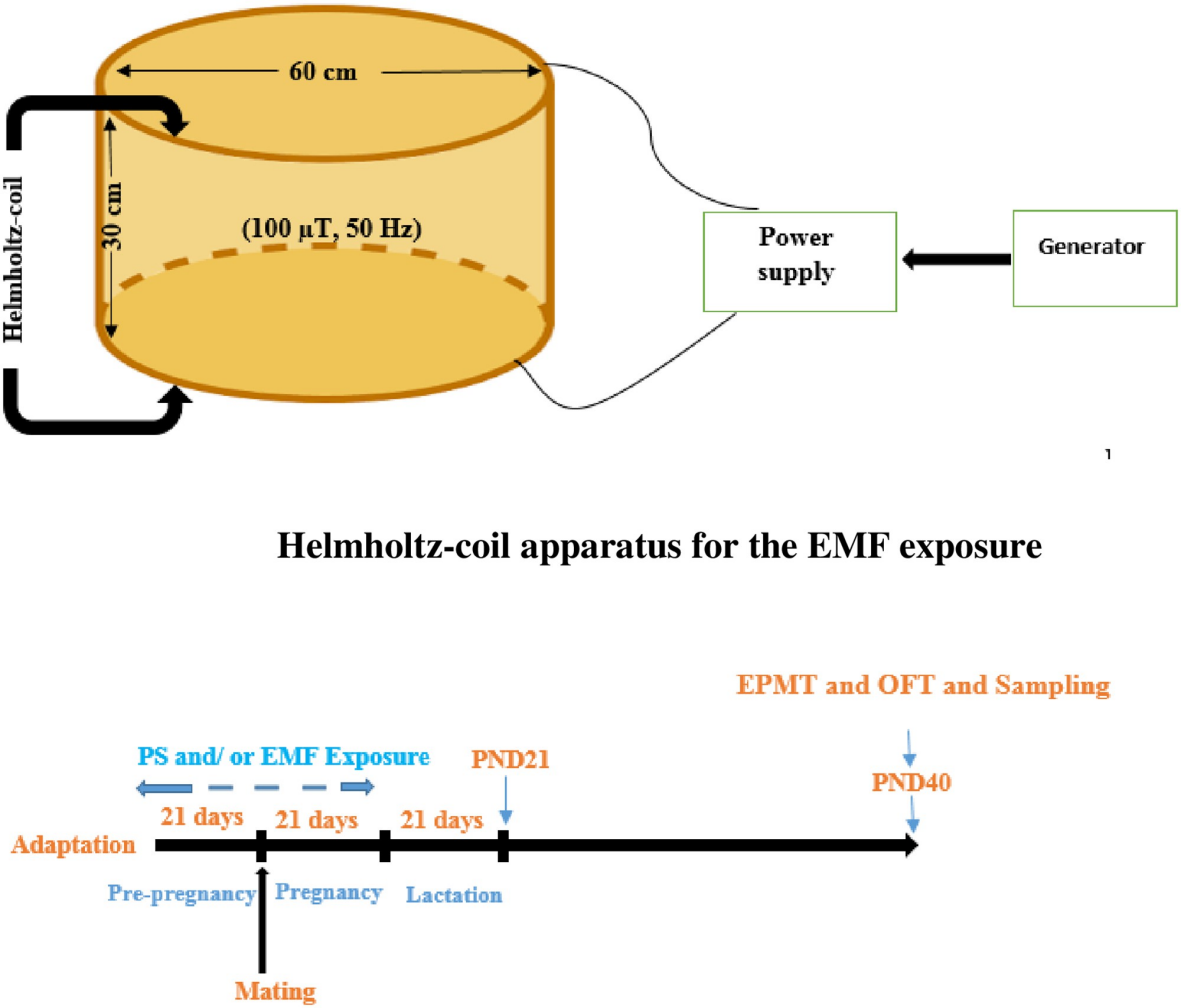
The experimental design of the study. PS: Prenatal stress, EMF: Electromagnetic field, PND: Postnatal day, EPMT: Elevated plus maze test, OFT: Open field test.

**Table 1:** Schedule of chronic stress.

Days	Light/darkness	Multiple Stress procedures timing
Sunday	Lights on overnight	Strobe light on 10.00 Room light off Untilt cages Strobe light off 16.00 Room lights on Soil bedding Start food and water deprivation
Saturday	Lights on overnight	Dry cage 11.00 Add water bottle Food ad libitum 12.00 Remove water bottle White noise on White noise off 15.00
Monday	Lights off overnight	Empty water bottle 10.00 Add water bottle 11.00 Strobe lights on 17.00
Tuesday	Lights off overnight	Strobe lights off 10.30 Remove food Tilt cages White noise on 15.30 Paired housing Untilt cages White noise off 23.30
Wednesday	Lights off overnight	Rehouse singly 10.00 Restricted food Add food ad libitum 12.00
Thursday	Lights on overnight	Start food and water deprivation 19.00
Friday	Lights on overnight	Restore food and water 14.30 Tilt cages

### 2.3 Stress procedure

The stress protocol of this study was according to Lewitus [58] with some modification. In the present study, the stress period was induced for 3 weeks before and 3 week of pregnancy. Three weeks of stress has been defined as chronic stress for rats in previous studies [58,59].

Each week the stress diet consisted of 8 different stress modes (Table 1), including: two periods of stroboscopic lighting (300 flash / min), one period of dry cage, two periods of noise (80 db), two periods of 45° cage tilt, one period of pair housing, and periods of water and food deprivation in which water deprivation was empty water bottles and food deprivation was followed by limited food intake [60].

### 2.4 Electromagnetic field device

The magnetic field device used in our study is based on Helmholtz’s screw theory designed in a physiology laboratory. The field-generating part of this machine consists of two coils with a radius of 30 cm located coaxially at 30 cm distance from one another. In the space between the two rings, a uniform field is created. The material of the rings containing coils is wooden and no metal was used in the mentioned distance, with a wooden tripod embedded on a sheet of foam. To produce a uniform field throughout this complex no metal parts were used. In this study the induced current was 50 Hz at an intensity of 100 μT [61].

### 2.5 Behavioral tests

#### Elevated plus-maze (EPM)

This tool is made of wood and has four arms arranged in the shape of a plus sign. Two of the arms have no side or end walls (open arms; 50×10 cm). The other two arms have side walls and end walls, but are open on the top (closed arms; 50×10×40 cm). To prevent rats from falling, a 1 cm height glass beam was installed on both sides and at the end of the open arms. The four arms lead to a central area of 10×10 cm. The maze is supported by stands at a height of 50 cm. The rats were placed within the central area of the maze facing an open arm. Suitable light is provided with a 100-watt bulb located at a height of 120 cm from the center of the maze. While doing the test, every rat was positioned in the central part so that it could move freely in different parts of the maze for 5 minutes. The following parameters were measured by observation: 1. The number of entries to the open arms. 2.The number of entries to the closed arms. 3. The total time spent in the open arms. 4. The total time spent in the closed arms 5. The percentages of open arm entries (OAE%). 6. The percentage of time spent in the open arms (open arm time, OAT%). These were calculated for each animal as follows: OAE%: (the ratio of entries into open arms to total entries×100); OAT%: (the ratio of times spent in the open arms to total times spent in any arms×100). OAE% and OAT% are defined as anxiety indices [62].

### 2.6 Open Field Tests (OFT)

This test was performed using a wooden compartment measuring 100 x 100 cm with a height of 30 cm. The inside of the chamber is divided into 25 equal square of 20 × 20 cm; The rat was then placed in the central square and the behavior was monitored for 5 minutes [63] as follows: 1. The number of times standing or leaning on the wall (one or two paws in contact with the wall). 2. The number of times rearing (standing on two hind paws without touching the walls). 3. The number of grooming behaviors (face cleaning, paw licking, fur licking, head scraping, and rubbing). 4. The number of defecations. 5. The number of center square entries (frequency with which the rat entered the center region with all four paws). At the end of each test, the box was cleaned with 70% alcohol and water to remove anything that could distract rat behavior in subsequent tests. All these experiments were recorded with a camera connected to the recording and analysis system. The system and the experimental instruments were located in an independent room to avoid the interference of artificial factors. For all behavioral tests, rats were brought to the testing room more than half an hour before the test.

### 2.7 Sampling

After the behavioral tests, rats were anesthetized with an intraperitoneal injection of ketamine hydrochloride and xylazine (60 and 12 mg / kg, respectively) [38]. The skulls of the rats were dissected using a guillotine, then the hippocampus were isolated and frozen at -80°C. All sampling was performed at 9:00 a.m. The right hemispheres were used for western blotting and the left hemisphere for ELISA.

### 2.8 Measurements of serotonin and 24(s) hydroxyl cholesterol

Serotonin, 24(s) hydroxyl cholesterol and corticosterone concentrations was determined in the hipocampous of all groups (n = 6) using the rat serotonin, 24(s)- hydroxyl cholesterol and corticosterone enzyme-linked immuno-specific assay (ELISA) kit (Elabscience, E-EL-003396, USA) (abcam, ab204530, USA)(abcam, KGE009, USA) respectively, following the manufacturer’s instructions. The absorbance of each well was measured at a wavelength of 450 nm using a 96-well microplate spectrophotometer (Awerness /stat Fax 4200). This assay can detect rat serotonin and 24(s)- hydroxyl cholesterol and corticosterone in the ranges of 15.63–1000 ng/mL and 0.39–100 ng/ml and 0.46–100 ng/mL respectively.

### 2.9 Western blot

The hippocampi were gently homogenized using a teflon homogenizer (Thomas) in 7 volumes of cold suspension buffer (20 mM HEPES-KOH (pH7.5), 250 mM sucrose,10 mM KCl, 1.5 mM MgCl2, 1Mm EDTA, 1 mM EGTA, 1 mM DTT, 0.1 mM PMSF, 2 mg/ml aprotinin, 10 mg/ml leupeptin, 5 mg/ml pepstatin, and 12.5 mg/ml of N-acetyl-Leu-Leu-Norleu-Al). Protein concentrations were determined with the BCA protein assay reagent (Pierce). Proteins (10 μg) were separated by SDS–PAGE (10% gel), followed by transfer to a polyvinylidene difluoride membrane (Millipore) and were blocked with 3% BSA in phosphate-buffered saline (PBS) for 1 h. The membrane was incubated overnight at 4°C with anti-STAR (sc-166821, 1:500), Anti- cryp (sc-293263, 1:200), Anti-3β-HSD (sc-515120, 1: 500), Anti- NMDA (ab14596, 1:500), Anti-PNMDA (ab16646, 1:250). The appropriate secondary antibodies were used to detect the anti-body antigen complex on membrane. GAPDH (Sigma) was applied as a gel loading control at a 1:500 dilutions. The obtained images were analyzed by image j then the density of each target protein band normalized to GAPDH corresponding band. The blots were developed with chemiluminescence detection system (Pierce ECL, Thermo Fisher Scientific). For developing, HyBlot CL (Denville Scientific, Metuchen, NJ) and Amersham Biosciences Hyperfilm were used to detect multiple proteins, membranes were stripped and then reprobed. Quantification of immunoblots was done using the UN-SCAN-IT software (Silk Scientific Inc., Orem, UT).

### 2.10 Statistical analysis

Normality of the data distribution was checked and confirmed using the Shapiro-Wilk test. All data are presented as mean ± SEM. The data was analyzed using SPSS 25 using two-way analysis of variance (ANOVA) with chronic stress and EMF as main fixed factors, followed by Tukey’s post-hoc test. P<0.05 was considered significant.

## 3. Results

### 3.1 The effect of EMF and prenatal stress on Corticosterone levels

**Fig 2:**
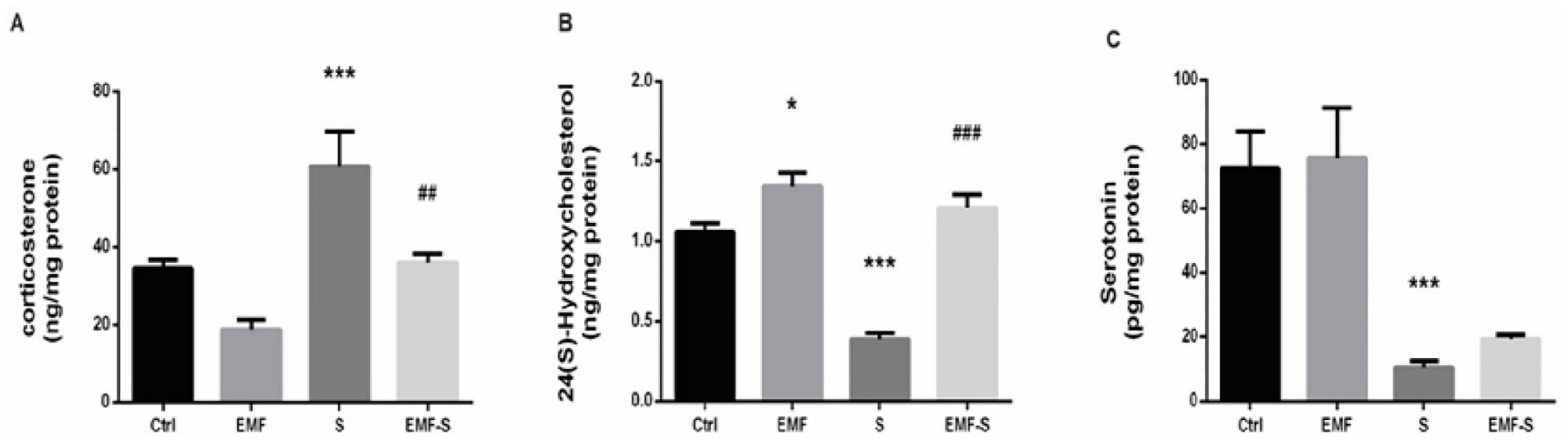
Effect of EMF on hippocampal neurostroids and serotonin levels after prenatal stress. Graphs show the level of (A) corticosterone, (B) 24(S)-OH cholesterol and (C) serotonin. Data are shown as mean ± SEM (n = 6 per group). Two‐way ANOVA, followed by Tukey’s post-hoc test: *p <0.05, ***p < 0.001 versus control group; ##p <0.001; ###p < 0.001 versus stress group. ANOVA, analysis of variance; Ctrl, Control; EMF, Electromagnetic Field; S, Stress.

Corticosterone did not show any statistically considerable change between EMF group (18.82±2.45) and control (34.53±2.16), (P = 0.136, F = 12.21). Corticosterone in stress group (60.7±8.99) significantly increased compared to control(34.53±2.16), (p < 0.001). EMF exposure decreased corticosterone level in EMF/S group (35.88±2.55) compared to Stress group (P<0.01). Also, EMF/S showed significant increase in comparison with EMF (P<0.01) (Fig 2A).

### 3.2 The effect of EMF and prenatal stress on 24(S)-OHC levels

As presented in Fig 2B, EMF exposure, increased the 24(S)-OHC of EMF group (1.34±0.08) compared to control (1.06±0.05), (p < 0.05), (p = 0.0352, F = 37.6). 24(S)-OHC significantly decreased in Stress group (0.38±0.03) compared to control (p <0.001). Also EMF/S group (1.21±0.08) showed increased 24(S)-OHC compared Stress group (p < 0.001). There was not any significant difference between EMF/S (1.21±0.08) and EMF (1.34±0.08) (P = 0.5377, F = 37.6) and also there was not any significant difference between EMF/S (1.21±0.08) and control (1.06±0.05), (P = 0.4322, F = 37.6) (Fig 2B).

### 3.3 The effect of EMF and prenatal stress on Serotonin content

Serotonin was significantly decreased in stress (10.53±1.89) compared to control(72.49±11.44), (p<0.001, P = 0.0006, F = 12.57) but no change was observed in EMF(75.77±15.49) compared to control (P = 0.9950, F = 12.57). It EMF/S (19.41±1.19), did not show any difference with Stress group (P = 0.91, F = 12.57). Also EMF/S showed significant decrease compared to EMF (p<0.001,P = 0.0017, F = 12.57) (Fig 2C).

### 3.4 The effect of EMF and prenatal stress on cryptochrome levels

**Fig 3:**
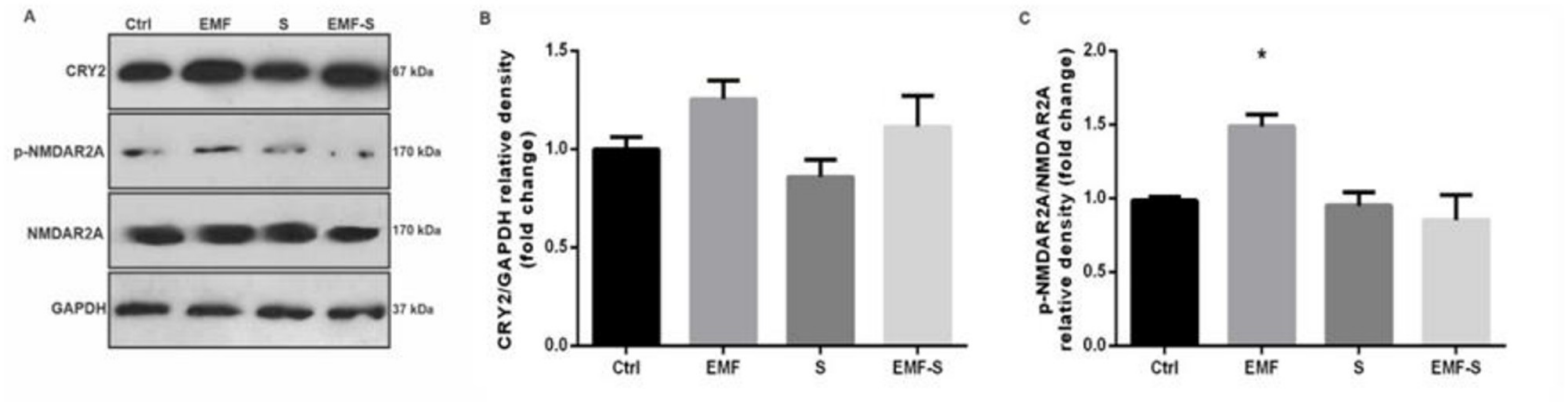
Effect of EMF on cryptochrome 2(cry2) and N-Methyl-D-Aspartate receptor 2(NMDAr2) / phosphorylated N-Methyl-D-Aspartate receptor2 (PNMDAr2) after prenatal stress. A representative immunoblotting image of cry2, NMDAr2, PNMDAr2 and GAPDH in different groups (A). Graphs show the protein expression of cry2 (B) and NMDAr2/ PNMDAr2 (C). Data are shown as mean ± SEM (n = 6 per group). Two‐way ANOVA, followed by Tukey’s posthoc test: *p < 0.05 versus control group; ANOVA, analysis of variance; Ctrl, Control; EMF, Electromagnetic Field; S, Stress.

The amount of cryptochrome 2 did not show any statistically difference among groups. (F = 2.514) (Ctrl vs EMF, P = 0.3814), (Ctrl vs S, P = 0.7891), (Ctrl vs EMF-S P = 0.8655), (EMF vs S, P = 0.1111), (EMF vs. EMF-S, P = 0.7888), (S vs EMF-S, P = 0.381) (Fig 3B).

### 3.5 The effect of EMF and prenatal stress on PNMDAr2/NMDAr2

PNMDAr2/NMDAr2 increased in EMF group (1.48±0.08) significantly increased compared to control (0.98±0.02), (P<0.05, P = 0.0342, F = 5.355). The other groups did not change significantly compared to control. There was significant difference between EMF/S (0.85± 0.16) and EMF (1.488 ± 0.08327), (F = 5.355, P<0.01, P = 0.0065) (Fig 3C).

### 3.6 The effect of EMF and prenatal stress on STAR levels

**Fig 4:**
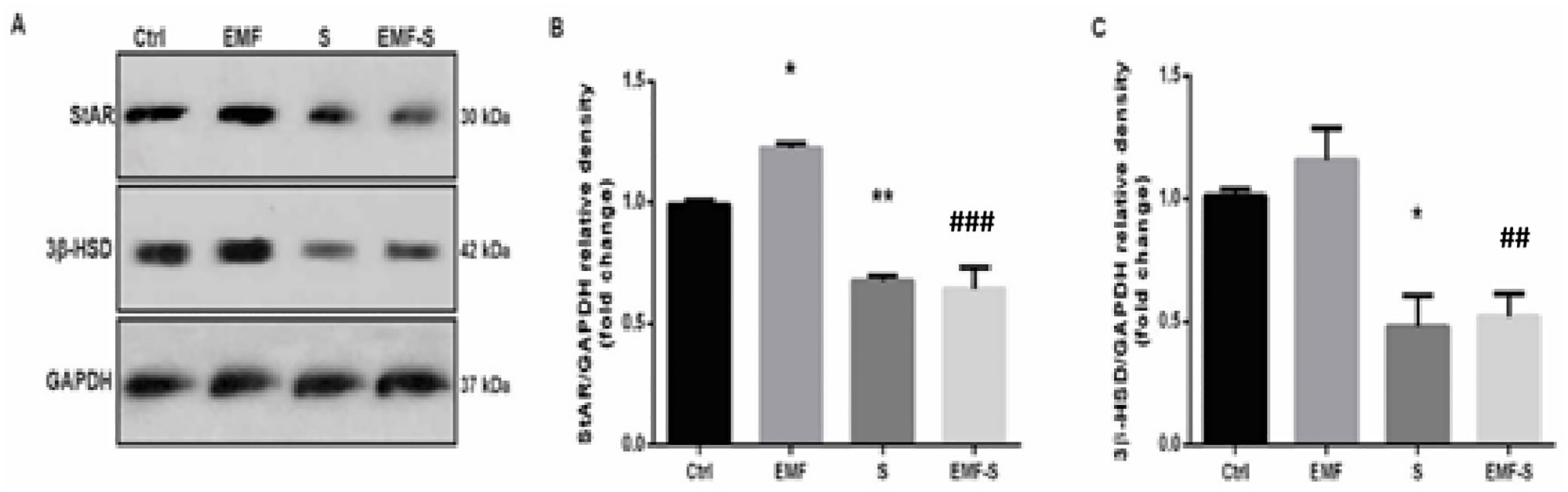
Effect of EMF exposure on steroidogenic acute regulatory protein (STAR) and 3B-Hydroxy steroid dehydrogenase (3β-HSD) expression after prenatal stress. A. representative immunoblotting image of STAR, 3β-HSD and GAPDH in different groups. Graphs show the quantified protein bands of (B) STAR and(C) 3β-HSD normalized to GAPDH and were presented as fold of control. Data are shown as mean ± SEM (n = 6 per group). Two‐way ANOVA, followed by Tukey’s posthoc test: *p < 0.05, **p < 0.01 versus control group; ## P< 0.01, ### P< 0.001 versus EMF; ANOVA, analysis of variance; Ctrl, Control; EMF, Electromagnetic Field; S, Stress.

STAR in EMF group (1.22±0.01) increased compared to control (0.99±0.02), (P<0.05, P = 0.0352, F = 32.79) and decreased meaningfully in Stress group 0.67±0.02) compared to control (p<0.01, P = 0.0076, F = 32.79). EMF/S (0.64±0.08) group did not show any statistically difference compared with Stress, (P = 0.96, F = 32.79). Also EMF/S showed significant decrease compared to EMF (p<0.001, P = 0.0001) (Fig 4B).

### 3.7 The effect of EMF and prenatal stress on 3B-HSD

3B-HSD in stress group (0.48±0.12) decreased significantly compared to control (1.01± 0.02), (P<005, P = 0.0266, F = 11.19), moreover, 3B-HSD of EMF group (1.15±0.12) did not change significantly in comparison with control, (P = 0.74, F = 11.19). EMF /Stress group (0.52±0.09) did not exhibit any significant difference with stress group, (P = 0.99, F = 11.19). Also EMF/S showed significant decrease compared to EMF (p<0. 01, P = 0.0098, F = 11.19) (Fig 4C).

### 3.8 The effect of EMF and prenatal stress on anxiety-like behavior (Elevated plus maze test)

**Fig 5:**
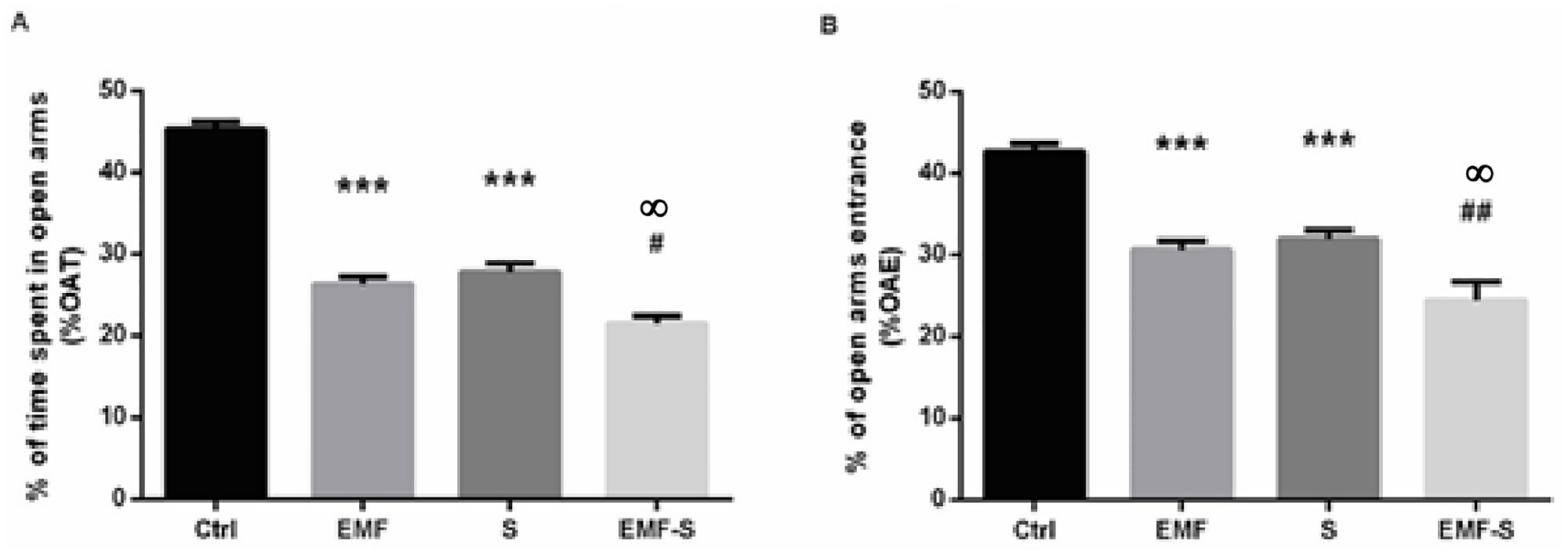
Effect of EMF on open arm time (%OAT) and open arm entries (%OAE) in plus maze test after prenatal stress. Graphs show the %OAT (A) and %OAE (B). Data are shown as mean ± SEM (n = 6 per group). Two‐way ANOVA, followed by Tukey’s post hoc test: ***p < 0.001 versus control group; #p<0.05, versus stress group; ##p <0.01; versus stress group; **∞** P<0.05, versus EMF. ANOVA, analysis of variance; Ctrl, Control; EMF, Electromagnetic Field; S, Stress.

Percentage of time spent in open arms, in EMF (26.33 ± 0.88) and Stress (27.83 ± 1.1) groups decreased compared to control (P<0.001) and in EMF/S group (21.50 ± 0.99) decreased compared to stress group (P<0.05, P = 0.032, F = 12.08). Also EMF/S showed significant decrease compared to EMF (p<0.05, P = 0.38) (Fig 5A).

Percentage of entrance in open arms of elevated plus maze, in EMF (30.67±1.11 P = 0.0006) and Stress (32±1.06, P = 0.0005) group significantly decreased compared to control (42.67± 1.02), (P<0.001) and in EMF/S group (24.5±2.23) statistically decreased compared to stress group (P<0.01, P = 0.0076, F = 13.52). Also EMF/S showed significant decrease compared to EMF (p<0.05, P = 0.036) (Fig 5B).

### 3.9 The effect of EMF and prenatal stress on anxiety-like behavior (Open field test)

**Fig 6:**
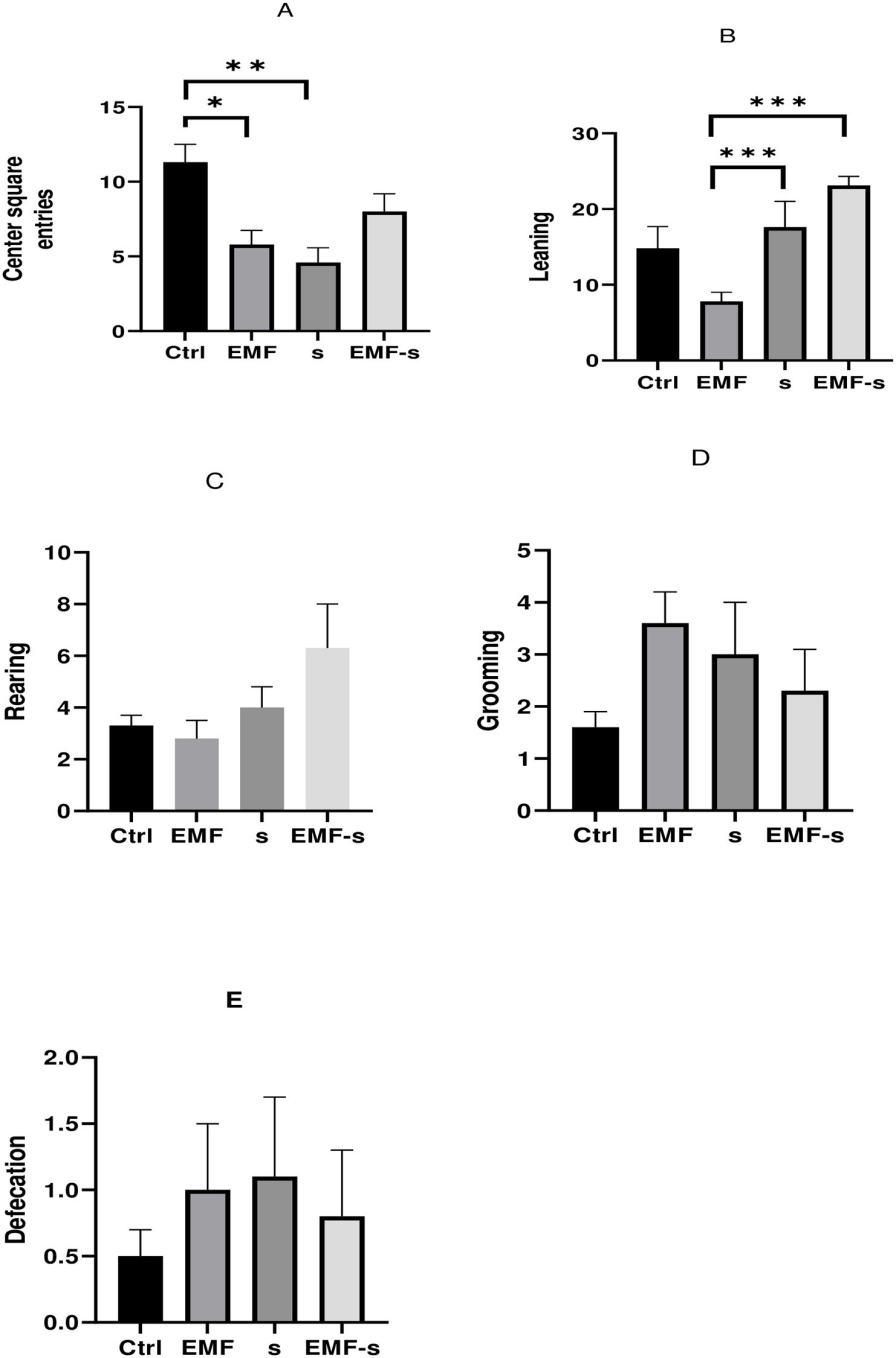
Effect of EMF on Center square entries (A), Leaning (B), Rearing (C), Grooming (D), Defecation (E), after prenatal stress. Data are shown as mean ± SEM (n = 6 per group). Two‐way ANOVA, followed by Tukey’s posthoc test: *p < 0.05, **p < 0.01, ***P<0.001, ANOVA, analysis of variance; Ctrl, Control; EMF, Electromagnetic Field; Stress, Prenatal stress.

Center square entries decreased in EMF (5.8±0.94, P = 0.29, F = 14.53) compared to control (11.3±1.2) and also Stress (4.6±0.98) group showed significant decrease compared to control (P <0.01, P = 0.008). Leaning in stress (17.6±3.4, P = 0.0007) and EMF/S (23.3±1.2, P = 0.0009) groups was higher than EMF group (7.8±1.2) (Fig 6).

## 4. Discussion

In the present study, we studied the effect of prenatal stress along with ELF-EMF on anxiety-like behavior in female offspring to assess possible alteration in neurostroids formation and metabolism, cryptochrome2 expression as a marker of circadian rhythm, glutamate receptor activation, and serotonin production in the hippocampus. We observed that although anxiety-like behavior, increased in all treatment groups, the EMF/S group showed more anxiety compared to both the ELF-EMF and Stress groups. For the first time, the present research found that prenatal stress combined with ELF-EMF brought more serious anxiety-like behaviors than prenatal stress alone or ELF-EMF alone in female rats. Furthermore we found elevated corticosterone levels in the Stress group, which can be one cause of the anxiety like behavior observed in this group. In addition, we observed a considerable serotonin decrease in the Stress group. It has been found that induction of stress during gestation causes changes in the central serotoninergic nervous system of offspring [64] and that prenatal maternal stress in mice results in a decrease in serotonin levels in offspring [65]. On the other hand, there are studies demonstrating that a decrease of serotonin levels in the hippocampus causes anxiety behavior in rats [66,67]. Hence, the anxiety-like behavior observed in the Stress group may be in relation to a decrease in hippocampal serotonin.

Conversely, elevated PNMDA r2/NMDA r2 (a criterion of NMDA receptor activation) and 24 (S) -hydroxy cholesterol levels in the ELF-EMF group indicated that anxiety-like behavior in this group could be attributed to these changes since studies have shown that activation of NMDA receptors (increase of PNMDAr/NMDA) in the hippocampus leads to anxiety-like behavior in rodents [36,68] and 24- (S) hydroxycholesterol is a positive allosteric modulator of NMDA receptors which may result in anxiety like behavior [69,70].

It should be noted that the hippocampus, amygdala and medial prefrontal cortex are anatomically and physiologically connected with projections to each other and cooperate together to initiate a relatively wide range of behaviors including anxiety behavior [71]. For example, CA1 forms the main output of the hippocampus [72] and projects to the medial prefrontal cortex and the amygdala [73,74], and in practice, CA1–prefrontal cortex inputs activate in anxiety-related behaviors [75] and activation of ventral CA1—basolateral amygdala synapses considerably increases anxiety-related behaviors [76,77]. It has been shown that corticosterone secretion following stress increases the excitability and firing of CA1 hippocampal pyramidal cells [78–80] which may be result in activation of ventral CA1—basolateral amygdala and CA1–prefrontal cortex circuitry and subsequently may thus initiate anxiety-like behavior, which could be an explanation for anxiety behavior seen in the Stress group of our study with noting to the increase of corticosterone in the stress group. Also, it has been found that NMDA receptors play a key role in generation of action potentials in CA1 [81], in a way that increases the activity of NMDA receptors (PNMDAr/NMDAr) which elevates the firing of CA1 cells that may stimulate ventral CA1–basolateral amygdala and CA1–prefrontal cortex circuitry. This may be a proximate cause of anxiety-like behavior observed in the EMF group in our study, considering increase in the PNMDAr/NMDAr ratio in the ELF-EMF group.

Cry2 is crucial for normal emotional behavior and is directly associated with anxiety-like behavior [82] and as mentioned earlier, mice with higher anxiety behavior exhibit lower expression levels of Cry2 in the hippocampus in comparison with normal mice [50]. Moreover it has been found that ELF-EMF (50–60 Hz) can disturb circadian biorhythms by perturbation of the clock function of cryptochromes [83]; however, we did not find any significant Cry 2 alteration in any of the treatment groups. It seems that further study is indicated to clarify the relationship between Cry2 and ELF-EMF exposure on one hand and between Cry2 and prenatal stress on the other.

In the present study, we assayed STAR and 3β-HSD enzymes to find the origin of possible changes in corticosterone levels. STAR is a key enzyme in the synthesis of corticosterone [45] and can affect production of corticosterone. In our study, we did not observe a direct relationship between the levels of STAR and corticosterone in the Stress and EMF groups. However, 3β-HSD, another crucial enzyme in synthesis of corticosterone, decreased significantly in the stress group compared with the control group, but, we did not observe a logical association between 3β-HSD enzymes and corticosterone levels in the Stress group.

## 5. Conclusion

**Fig 7:**
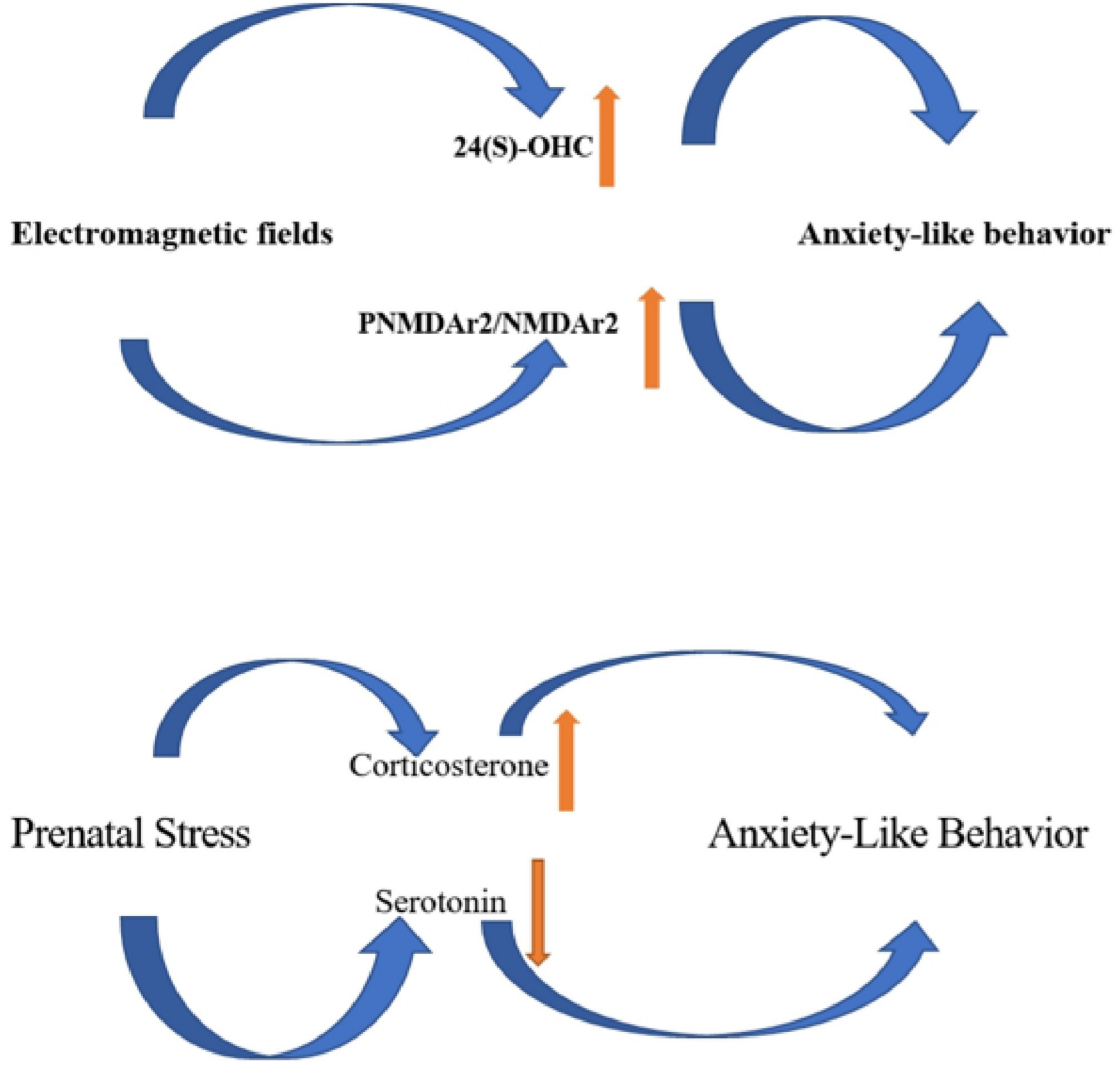
Summary of how ELF-EMF and prenatal stress could be able to induce anxiety-like behavior in offspring by modification of neurostroids metabolism, serotonin and N-Methyl-D-Aspartate receptor 2(NMDAr2) in hippocampus. ↑ indicating increasing effect, ↓ indication decreasing effect.

It may be concluded that involvement of ELF-EMF and prenatal stress in potential induction of anxiety-like behavior via the hippocampus may be different such that ELF-EMF may initiate anxiety-like behavior by increasing 25(S)-OHC and PNMDAr2/NMDAr2 in the hippocampus while prenatal stress probably increases anxiety-like behavior by elevating corticosterone and decreasing serotonin (Fig 7).

### Study limitations

There were some limitations in present study which could help clarify the findings, including lack of electrophysiological studies to find the effects of changes of the studied factors on hippocampal-amygdala-prefrontal cortex synapses and circuitry and lack of assessment of changes of factors such as corticosterone and NMDA receptors and so on in other regions involved in anxiety behavior such as the amygdala.

### Ethics approval and consent to participate

This project was approved by the Tabriz University of Medical Sciences Board. All procedures were performed in strict accordance with the international guidelines for care of experimental animals.

## Supporting information

S1 Dataset (DOCX)

S1 Raw images (PDF)
