# Supplementary material for: Maternal stress induced anxiety-like behavior exacerbated by electromagnetic fields radiation in female rats offspring
Source: PLoS One. 2022 Aug 23;17(8):e0273206. doi: 10.1371/journal.pone.0273206 (PMC9397925; doi:10.1371/journal.pone.0273206)
Supplement: S1 Dataset — (DOCX) [file pone.0273206.s001.docx]

**All data were written as Mean ± SE**

|  | Control | EMF | Stress | EMF/Stress |
| --- | --- | --- | --- | --- |
| **Percentage of time spent in open arms(****%OAT)** | 45.33 ± 0.95 | 26.33 ± 0.88 | 27.83 ± 1.1 | 21.50 ± 0.99 |
| **Percentage of entrance in open arms(****%OAE)** | 42.67 ± 1.02 | 30.67 ± 1.11 | 32 ± 1.06 | 24.5 ± 2.23 |

**Plus Maze Test**

**Open field Test**

|  | Control | EMF | Stress | EMF/Stress |
| --- | --- | --- | --- | --- |
| **Center square**  **entries** | 11.3±1.2 | 5.8±0.94 | 4.6±0.98 | 8±1.2 |
| **Leaning** | 14.8±2.9 | 7.8±1.2 | 17.6±3.4 | 23.3±1.2 |
| **Rearing** | 3.3±0.4 | 2.8±0.7 | 4±0.8 | 6.3±1.7 |
| **Grooming** | 1.6±0.3 | 3.6±0.6 | 3±1 | 2.3±0.8 |
| **Defecation** | 0.5±0.2 | 1±0.5 | 1.1±0.6 | 0.8±0.5 |

|  | Control | EMF | Stress | EMF/Stress |
| --- | --- | --- | --- | --- |
| **Corticosterone**  **(ng/mg protein)** | 34.53±2.16 | 18.82±2.45 | 60.7±8.99 | 35.88±2.55 |
| **24(S)-OH cholesterol**  **(ng/mg protein)** | 1.06±0.05 | 1.34±0.08 | 0.38±0.03 | 1.21±0.08 |
| **Serotonin**  **(Pg/mg protein)** | 72.49±11.44 | 75.77±15.49 | 10.53±1.89 | 19.41±1.19 |

**ELISA Tests**

**Western Blot Tests**

|  | Control | EMF | Stress | EMF/Stress |
| --- | --- | --- | --- | --- |
| **Cryptochrome/GAPDH relative Density(Fold Change)** | 1±0.06 | 1.25±0.09 | 0.86±0.08 | 1.11±0.15 |
| **PNMDAr2/ NMDAr2 relative Density(Fold Change)** | 0.98±0.02 | 1.48±0.08 | 0.95±0.09 | 0.85±0.16 |
| **STAR/GAPDH relative Density(Fold Change)** | 0.99±0.02 | 1.22±0.01 | 0.67±0.02 | 0.64±0.08 |
| **3B-HSD/GAPDH relative Density(Fold Change)** | 1.01± 0.02 | 1.15±0.12 | 0.48±0.12 | 0.52±0.09 |
