# Supplementary material for: Maternal stress induced anxiety-like behavior exacerbated by electromagnetic fields radiation in female rats offspring
Source: PLoS One. 2022 Aug 23;17(8):e0273206. doi: 10.1371/journal.pone.0273206 (PMC9397925; doi:10.1371/journal.pone.0273206)
Supplement: S1 Raw images — (PDF) [file pone.0273206.s002.pdf]

ORIGINAL IMAGES FOR BLOTS AND GELS Original file

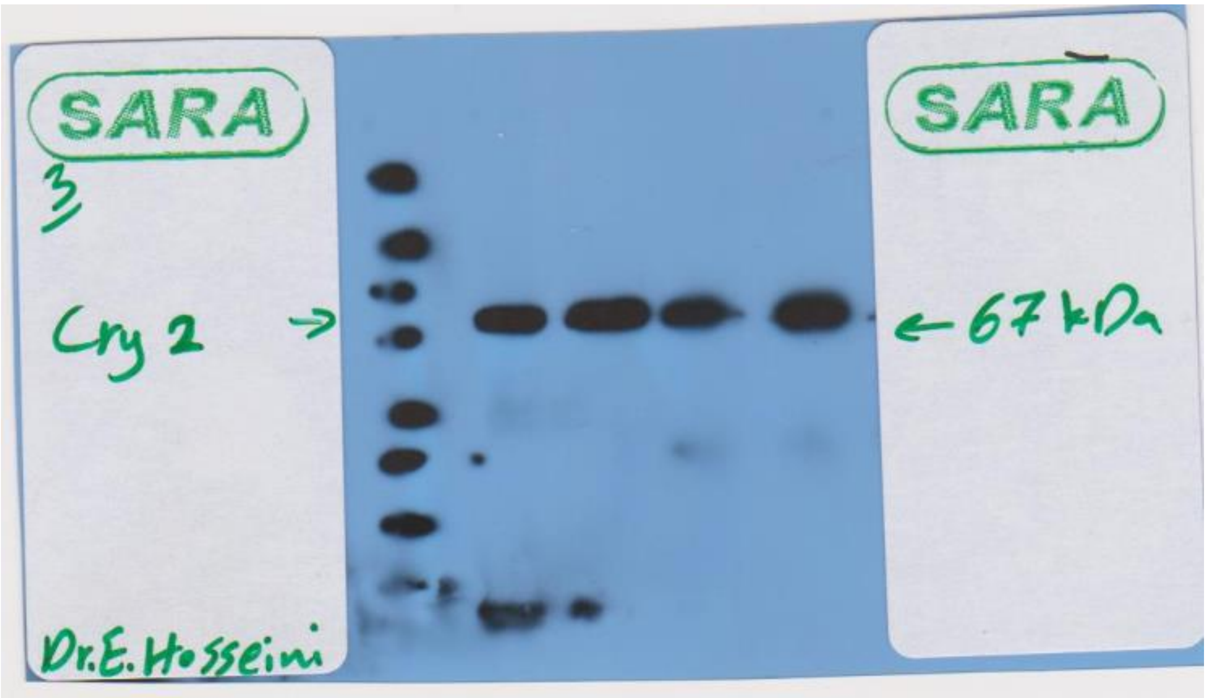

Fig3.original blot of cry2

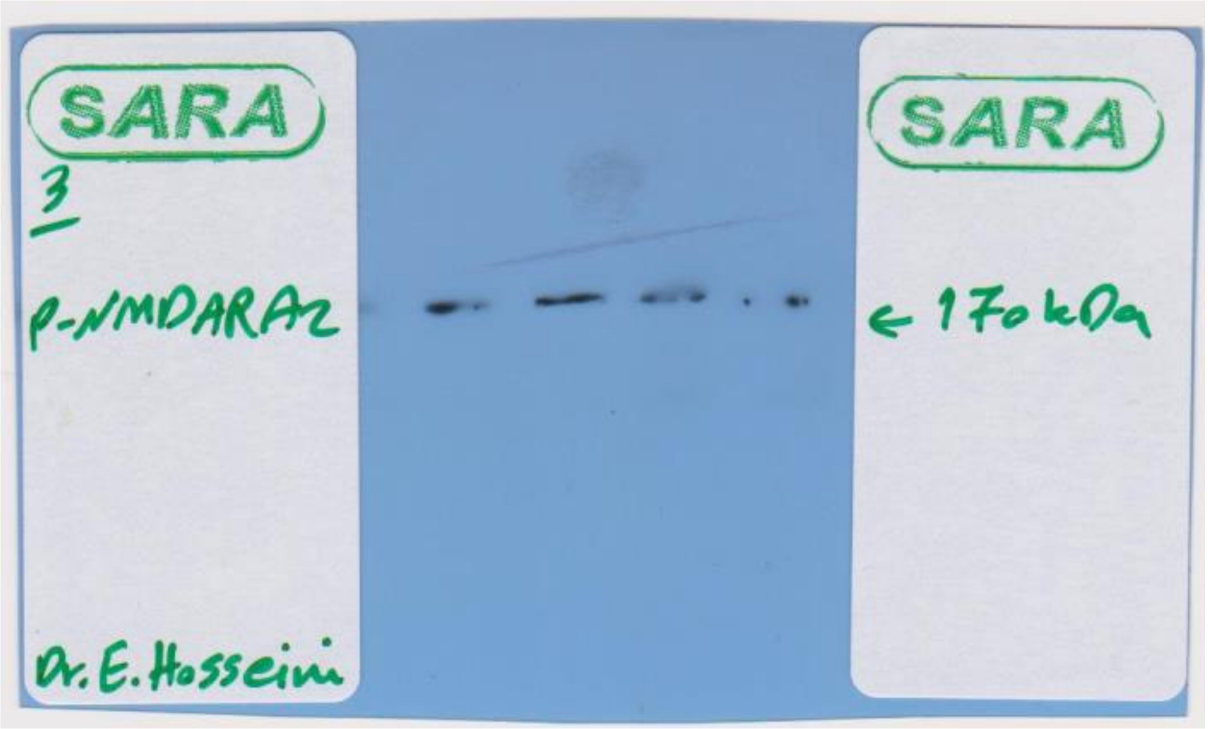

Fig3. Original blot of P-NMDAr2

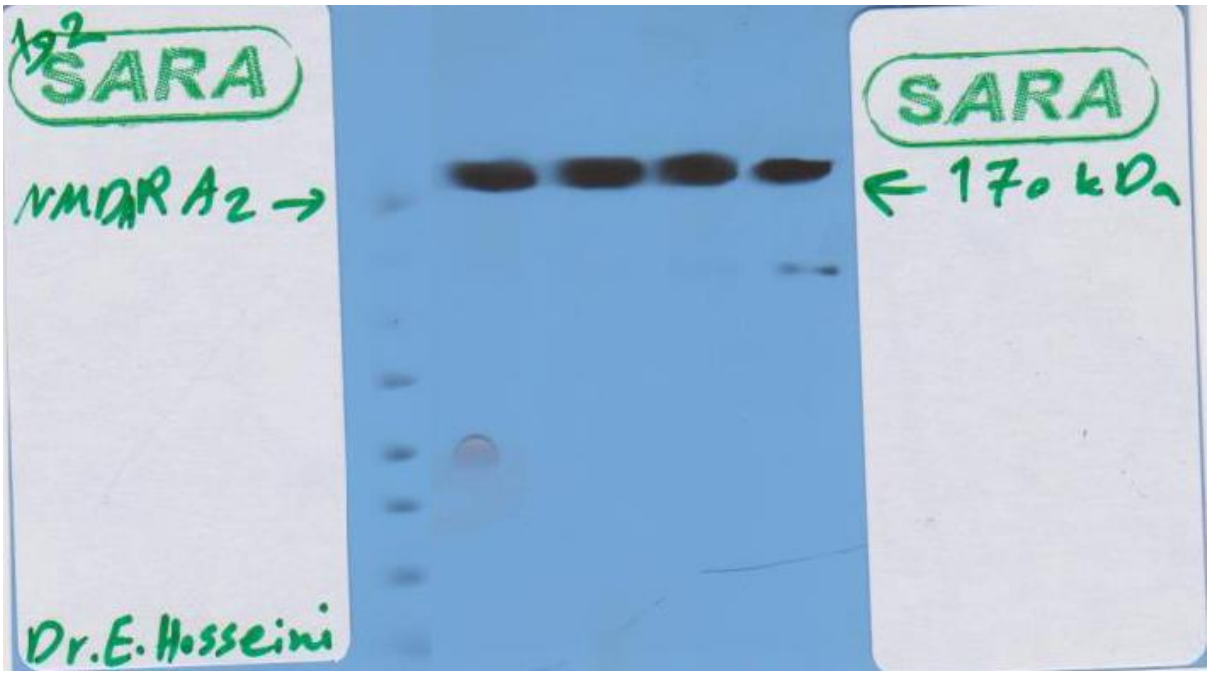

Fig3. Original blot of NMDAr2

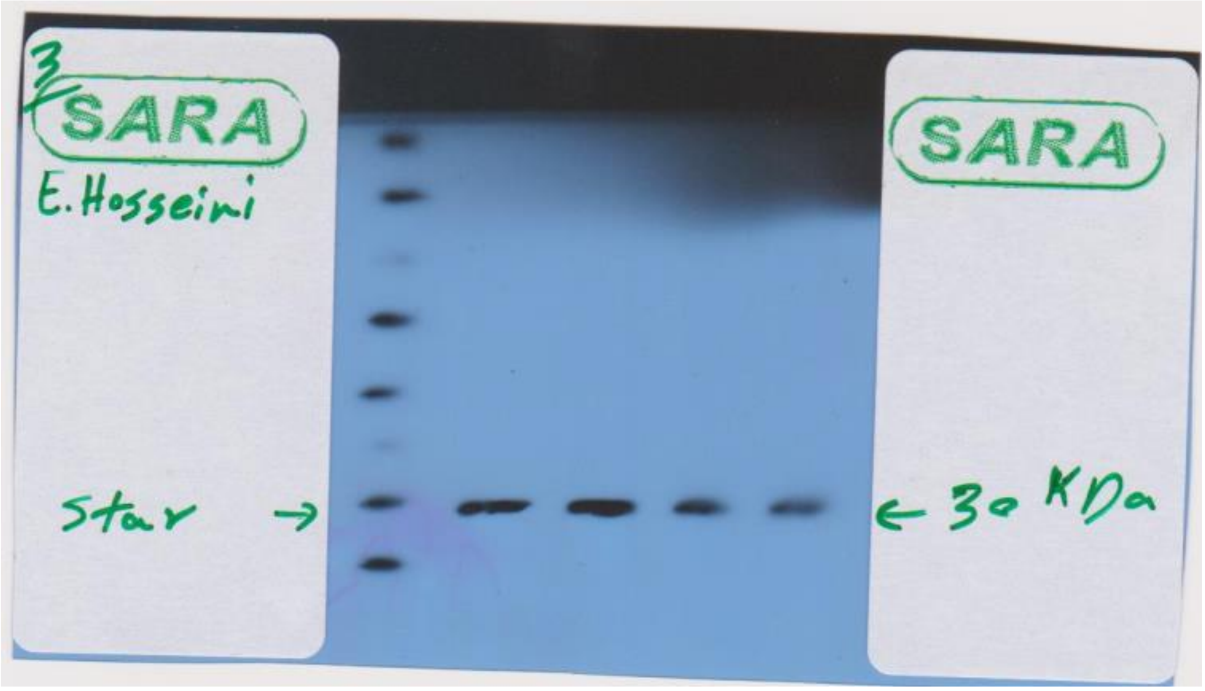

Fig4. Original blot of STAR

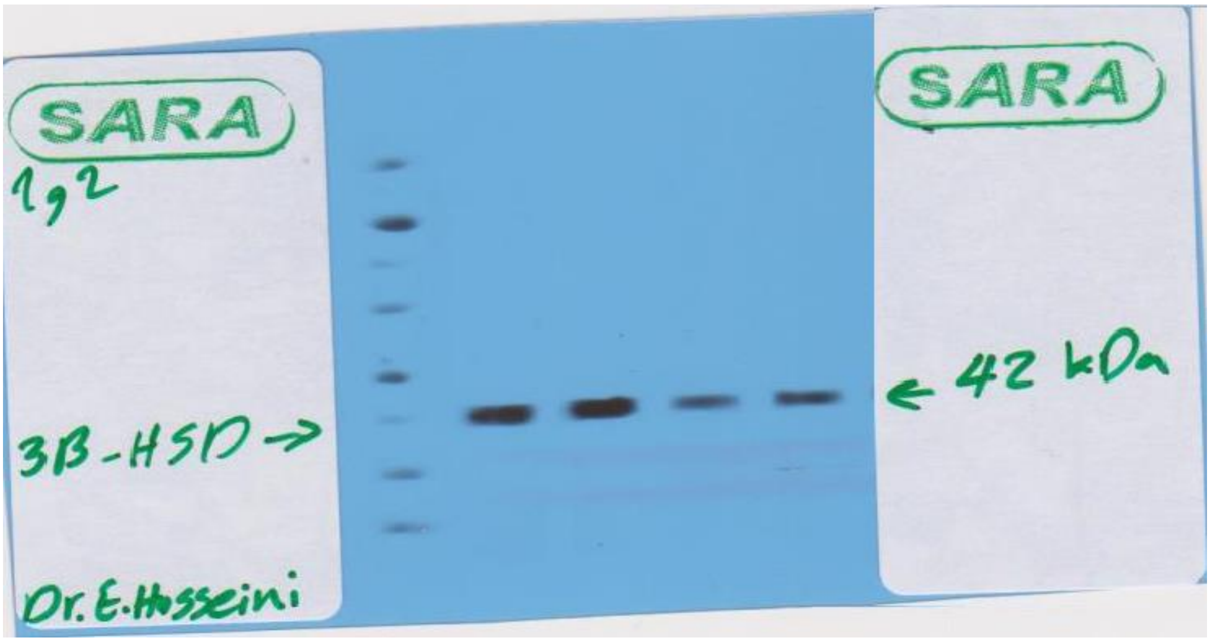

Fig4. Original blot of 3B-HSD

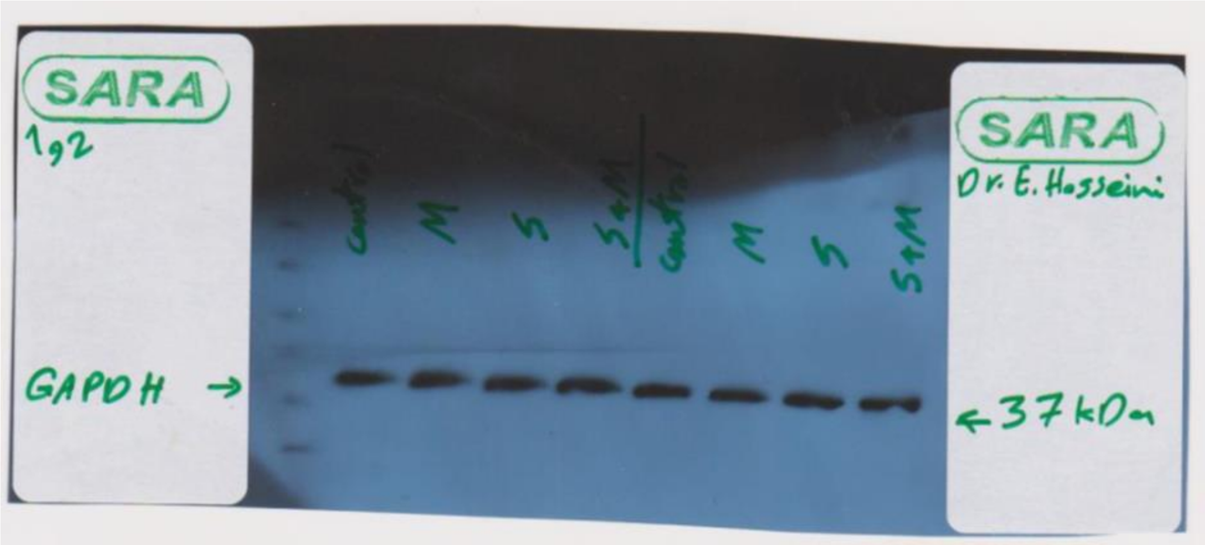

Original blot of GADH (the right row was selected)
